# The costs of bipolar disorder in the United Kingdom

**DOI:** 10.1002/brb3.2351

**Published:** 2021-09-15

**Authors:** Judit Simon, Anees A. Abdul Pari, Jane Wolstenholme, Michael Berger, Guy M. Goodwin, John R. Geddes

**Affiliations:** ^1^ Department of Health Economics, Center for Public Health Medical University of Vienna Vienna Austria; ^2^ Department of Psychiatry University of Oxford and Oxford Health NHS Trust Oxford United Kingdom; ^3^ HERC, Nuffield Department of Population Health University of Oxford Oxford United Kingdom; ^4^ Public Health England London United Kingdom

**Keywords:** bipolar disorder, cost, cost‐of‐illness, economic burden, productivity loss, United Kingdom

## Abstract

**Objectives:**

To estimate the individual cost and population‐level economic burden of Bipolar Disorder (BD), and explore the impact of clinical and sociodemographic factors on costs in the United Kingdom.

**Methods:**

Annual UK health care, social care and societal cost data were collected from a prospective cohort of 91 BD patients using digital monitoring of symptoms. Costs (in £) were calculated for the year of resource use collection (2010–2011) and main results inflated to year 2018–2019. A Generalized Estimating Equation framework was used to investigate individual factors influencing costs. An economic burden estimate was derived by multiplying the mean annual cost per patient with literature‐based population prevalence.

**Results:**

The average annual cost of BD per patient was £12,617 (SE = ±£1085) or £14,938 (SE = ±£1281) at 2018–2019 prices with 68% of the total costs attributed to lost productivity and informal care, 31% to health care costs, 1% to private out‐of‐pocket expenses, and 0.5% to social care costs. A unit increase in average levels of depressive or manic symptoms were associated with 7% and 11% higher societal costs, respectively. The estimated annual prevalence of BD in the United Kingdom was 0.8% resulting in a population‐level economic burden estimate of £5.1 billion for 2010–2011 or £6.43 billion for 2018–2019.

**Conclusions:**

BD is a disease of substantial costs in the United Kingdom with the majority of the economic burden falling outside the health care system in the form of productivity losses and informal care. These costs highly correlate with patient outcomes highlighting further needs for improved treatment efforts into functionality.

## Graphical abstract

We assess the individual cost and population‐level economic burden of Bipolar Disorder (BD), and explore the impact of clinical and sociodemographic factors on costs in the United Kingdom. Based on a prospective cohort of 91 BD patients, we estimate an annual prevalence of BD in the United Kingdom of 0.8% and average annual costs of BD £12,617 per patient in 2010–2011, resulting in a population‐level economic burden estimate of £5.1 billion for 2010–2011 or £6.43 billion adjusted to 2018–2019 prices. We further provide a breakdown of the annual costs per major cost categories.

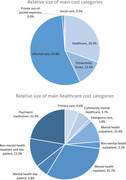

## 1 INTRODUCTION

Bipolar disorder (BD) is a debilitating chronic mental illness characterized by a remitting/relapsing course with episodes of mania and depression. The prevalence estimates of BD vary widely between studies; reviews have suggested estimates ranging from 0.5% to as high as 6.0% (Fajutrao et al., 2009; Ferrari et al., 2011; Pini et al., 2005). The recurrence rate for an affective episode rises to 50% by the second year increasing to a risk of 73% by 5 years postdiagnosis. There is no completely satisfactory therapy targeting underlying disease mechanism, although there are several reasonably effective treatments for the acute illness and for preventing relapse (Geddes & Miklowitz, 2013; Kroon et al., 2013; Perlis et al., 2006). BD leads to significant impairment of health‐related quality of life (HRQOL) and well‐being along with detrimental emotional, financial and health implications not only for the patients, but also their families and the wider society (Ishak et al., 2012). However, there is less knowledge of the total disease burden associated with BD in the United Kingdom. Estimating the costs of BD can provide valuable insights into its relative burden and cost distribution along with the opportunity to explore factors that have the greatest impact on health, social care and societal resource utilization. This evidence may be critical in raising awareness of the disease or aspects of the disease, thereby contributing to discussions and decisions around efficient resource allocation (Larg & Moss, 2011).

Few studies have comprehensively assessed the economic burden of BD in Europe, and only three studies have attempted to estimate it in the United Kingdom according to the latest international review (see Appendix Table A1) (Jin & McCrone, 2015).

The two previous economic burden assessments for BD in the United Kingdom (Das Gupta & Guest, 2002; McCrone et al., 2008) are based on data that are more than 20 years old, whereas the more recent study by Young et al. (2011) for the year 2007–2008 only considered health care‐related costs, which previous studies showed to represent only a minor fraction of the total economic burden. All three UK‐based studies gave very different health care cost estimates, most likely due to the use of divergent prevalence estimates for BD and the variance in the applied study methodologies (see Table 1).

**TABLE 1:** Baseline participant characteristics

Characteristics of participants	OXTEXT‐2 (*n* = 91)	Complete cases (*n* = 48)	OXTEXT‐1 (*n* = 271)
Mean age in years (SEM, range)	45.5 (1.5, 20–75)	51 (1.7, 20–74)	40.4 (0.8, 17–76)
Female gender, *n* (%)	58 (63.7%)	33 (68.8%)	184 (67.9%)
Ethnicity, *n* (%)			
Caucasian	88 (96.7%)	46 (95.8%)	256 (94.5%)
Non‐Caucasian	3 (3.3%)	2 (4.2%)	15 (5.5%)
Employment status, *n* (%)			
Employed/self‐employed	45 (49.5%)	25 (52.1%)	136 (50.2%)
In education	8.0 (8.8%)	3 (6.3%)	33 (12.3%)
Unemployed/retired	38 (41.8%)	20 (41.7%)	102 (37.5%)
Mean years of education (SEM, range)	15.8 (0.31, 11–19)	15.9 (0.47, 11–19)	15.9 (0.16, 7–19)
Diagnosis, *n* (%)			
BD‐I	55 (60.4%)	31 (66.0%)	171 (63.1%)
BD‐II	36 (39.6%)	17 (36.0%)	100 (36.9%)
Mean baseline QIDS score (SEM, range)	8.2 (0.51, 0.0–22)	6.9 (0.79, 2.0–22)	9.9 (0.40, 0.0–24)
Mean baseline ASRM score (SEM, range)	3.0 (0.34, 0.0–16)	3.3 (0.59, 0.0–16)	3.5 (0.31, 0.0–20)

Over the past decade there has been an emergence of new therapies (e.g., second generation antipsychotics), modalities of care (e.g., adjunct psychotherapies) and service delivery options (e.g., collaborative care, reduced admission beds) (Geddes & Miklowitz, 2013). Notwithstanding the high variation of the results of the three existing UK‐based cost‐of‐illness studies measuring the population‐level economic burden caused by BD owing to their methodical heterogeneity, it is likely that all of them actually underestimate the current economic burden of BD in the United Kingdom on the counts of being based on older and outdated therapy regimens and care pathways. Therefore, a more up‐to‐date cost‐of‐illness study both in terms of data and methodology following current good practice recommendations is required (Kleine‐Budde et al., 2014).

This study seeks to (a) assess the cost of BD over a 1‐year period through prospectively following‐up a well‐defined cohort of community BD patients in the United Kingdom, (b) explore the main clinical and sociodemographic factors that drive variations in the costs associated with BD, and (c) estimate the population‐level economic burden for the United Kingdom.

## 2 METHODS

### 2.1 Study population

The study used data obtained from the OXTEXT‐2 substudy which was part of the OXTEXT research programme (RP‐PG‐0108‐10087) and followed a cohort of 91 BD patients over a 12‐month period in 2010–2011. Patients were invited to take part between May 2010 and June 2011 from the larger OXTEXT‐1 cohort study which was a pragmatic longitudinal cohort of patients with a confirmed diagnosis of BD who attended psychiatric clinics in Oxford Health NHS Foundation Trust (OHFT) and were monitored with a user friendly SMS or web‐based system called “True Colours” (TC) (Goodday et al., 2020).

Eligible criteria were: males and females aged over 16 years old, with a primary diagnosis of BD according to the DSM‐IV‐R criteria, who were willing and able to provide informed consent to participation in the study. The study received ethical approval by the local Research Ethics Committee (Oxfordshire REC A, Reference: 10/H0604/13).

Clinical status of patients was measured using the five‐item Altman Self‐Rating Mania (ASRM) scale and 16‐item Quick Inventory of Depressive Symptoms–Self Report (QIDS‐SR) scale. The ASRM scale (scores range from 0 to 20) consists of five questions marked between 0 and 4 with increasing severity. In the literature, cut‐off point of less than or equal to 5 is indicative of “being not in a manic state,” whereas a score of greater than 5 indicates a “state of mania” (Altman et al., 1997). The QIDS‐SR consists of 16 items each scored between 0 and 3. Scores range from 0 to 27 and can be categorized as (a) no depression: 0–5, (b) mild depression: 6–10, (c) moderate depression: 11–15, (d) severe depression: 16–20, (e) very severe depression: 21–27 (Rush et al., 2003).

### 2.2 Data collection

The data on the use of health and nonhealth care resources were collected using self‐reported patient questionnaires based on 3‐month recall at four different time points: 0‐ to 3‐, 4‐ to 6‐, 7‐ to 9‐, and 10‐ to 12‐month follow‐ups. In addition to baseline information on sociodemographics, disease status and illness duration, data were collected on contacts made with primary, secondary, tertiary and social care; psychiatric medication; patients’ out‐of‐pocket expenses (e.g., private dietician, acupuncture); and productivity losses. Data on distance travelled to psychiatric health care providers and the time informal carers (e.g., family members and friends) spent providing unpaid care and support was also collected for each study participant.

### 2.3 Cost estimation

We used both health and social care, and societal perspectives in our analyses. Resource use categories were multiplied by their relevant national‐level unit costs using British pounds sterling (£). Unit costs were obtained from various sources including the National Schedule of Reference Costs (Department of Health, 2011), Unit Costs of Health and Social Care (Curtis, 2012), and the British National Formulary 65 for the original year of resource use collection (2010–2011) (Joint Formulary Committee, 2013) (see Appendix Table A2).

Lost productivity costs were estimated using the human capital approach (HCA). Days off work were multiplied by the average daily cost of sickness absence in the United Kingdom (CMH, 2007; NICE, 2009; Tarricone, 2006). These costs were estimated only for those who were employed or self‐employed (*n* = 53, 58.2%). Informal care costs were estimated by multiplying the average hourly salary derived from the average annual gross UK wage rate with the number of hours of unpaid work (Bovill, 2013; Faria et al., 2012).

Average annual per patient costs and population‐level economic burden were updated for year 2018–2019. For health care‐related costs, we used the Hospital & Community Health Services (HCHS) index for the period 2011–2012 to 2014–2015 and the NHS Cost Inflation Index (NHSCII) from 2015–2016 onward (Curtis & Burns, 2019), amounting to an increase of 18.05% over the entire inflation period. For social care costs, we used the Personal Social Services (PSS) pay and prices index for social care costs (Curtis & Burns, 2019), amounting to an increase of 14.27%. For lost productivity and informal care costs, we used the UK consumer‐price index (CPI) (ONS, 2020a) amounting to an increase of 18.59%.

### 2.4 Statistical analysis

A *p*‐value of ≤ .05 was used in all hypothesis tests of statistical significance. All statistical analyses were undertaken using STATA 12.0 (StataCorp, College Station, TX, USA).

Multiple imputation (MI) by chained equations was used with age, gender, type of BD, duration‐of‐illness, concurrent clinical measures, and the value of the missing variable at baseline as covariates (Schafer & Graham, 2002). The MI by chained equations procedure (“MI impute”) with predictive mean matching to account for skewed distribution in cost categories was applied to perform 100 imputations (MacNeil Vroomen et al., 2016).

To determine the influence of major sociodemographic and clinical characteristics such as age, gender, type of BD diagnosis, duration of illness and clinical status on overall costs and other major cost categories, Generalized Estimating Equation (GEE) was implemented on the fully imputed dataset. A multivariate regression model with gamma distribution and log‐link function was used to address skewed cost data and an exchangeable covariance matrix was found appropriate to account for the correlation between repeated covariates and their influence on the primary outcomes which were measured on multiple occasions (Raikou & McGuire, 2012). The final model was selected based on lowest quasi‐likelihood under the Independence model Criterion (QIC score) (Pan, 2001).

### 2.5 Population‐level economic burden

The cost‐of‐illness of BD in the United Kingdom was estimated as the product of the total mean annual societal cost per BD patient multiplied by the population prevalence of BD. In the absence of a UK population prevalence estimate for the initial study period, a rapid literature review was undertaken to synthesize 12‐month prevalence estimates of BD relevant for the United Kingdom. Embase and Medline were searched for studies published between 1990 and November 2013. The search was limited to Northern and Western European countries as defined by the United Nations Development Statistical Division (UN, 2013). Other inclusion criteria were (a) population or community‐based studies using random sampling methods, (b) cohort of people aged ≥16 years, and (c) studies in any of the languages spoken in the defined geographical area. Exclusion criteria were (a) studies using clinical samples and (b) studies using a narrow age band (<10 years). A pooled prevalence estimate was derived using the weighted inverse variance DerSimonian‐Laird method; the *I* ^2^ test was used to assess heterogeneity. We adjusted for population growth in the updated estimates by applying the prevalence rate of the study period to the mid‐2018 UK population estimate provided by the Office for National Statistics (ONS, 2019).

### 2.6 Sensitivity analyses

A series of one‐way sensitivity analyses were performed to explore how uncertainties in key input parameters impact the economic burden results for the original costing year 2010–2011. Cost estimates were varied according to their lower and upper 95% confidence intervals (CI). To check the impact of joint uncertainty in the input data and assumptions, a multivariate probabilistic sensitivity analysis (PSA) using Monte Carlo simulation was undertaken using Microsoft Excel 2010 (Claxton et al., 2005). In addition, a complete case analysis was carried out to compare the results of the imputed dataset with those of a complete case scenario. Sensitivity analysis results were not further inflated to year 2018–2019, since this step would have introduced only additional uninformative methodological uncertainties.

## 3 RESULTS

### 3.1 Study population

A total of 91 patients completed the baseline questionnaire. Follow‐up resource use questionnaires were available for 85% (77/91), 78% (71/91), 76% (69/91), and 75% (68/91) of the cohort at 3‐, 6‐, 9‐, and 12‐month follow‐ups, respectively.

Baseline characteristics of the full OXTEXT‐2 study sample (*n* = 91) alongside the characteristics of the complete cases (*n* = 48) are provided in Table 1, 2. The baseline characteristics of the included participants were similar to those of the original OXTEXT‐1 cohort (Table 1). Most of the participants were female (58/91, 64%), the baseline average age was 45.4 years (SE = ±1.46, 95% CI = 42.5–48.3), 42% (38/91) of the cohort were either unemployed or retired. The average duration of illness was 14 years (SE = ±1.13). Overall, 60% (55/91) of the patients met BD‐I criteria, 40% (36/91) were classified as BD‐II. The mean baseline QIDS score for depression was 8.2 (SE = ±0.51), the mean baseline ASRM score was 3 (SE = ±0.34). No episodes of self‐harm, completed suicides or deaths were recorded during the study period.

### 3.2 Cost estimates

**FIGURE 1:**
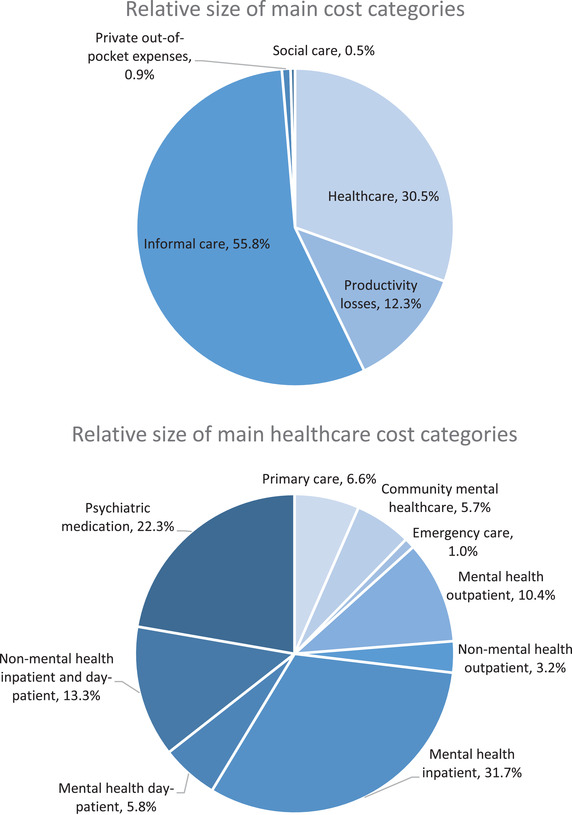
Distribution of the total societal costs and health care costs of BD by major categories

The mean annual societal cost of BD per patient was estimated at £12,617 (SE = ±£1085, 2018–2019 prices: £14,938). Costs by resource use category and their relative contributions to the economic burden are presented in Table 2 and Figure 1, respectively.

**TABLE 2:** Mean annual costs per BD patient (in £) based on imputed full sample (*n* = 91) and complete case analysis (*n* = 48)

Resource use categories	Imputed full sample (*n* = 91)				Complete case analysis (*n* = 48)			
Primary care costs	Mean	95% CI		Price‐adjusted mean^†^ (2018–2019)	Mean	95% CI		Price‐adjusted mean^†^ (2018–2019)
General practitioner	223.1	198.1	248.2	263.4	217.7	152.6	282.7	257.0
General practice nurse	30.2	25.3	35.0	35.7	34.0	21.1	47.0	40.1
Community mental health care costs								
Community psychiatric nurse	63.7	48.2	79.1	75.2	59.9	24.4	95.5	70.7
Community psychologist	37.6	21.9	53.2	44.4	21.8	–3.3	46.8	25.7
Community psychiatrist	54.2	36.2	72.2	64.0	39.8	14.3	65.3	47.0
Community drop‐in center	10.6	6.0	15.1	12.5	9.2	0.0	18.3	10.9
Support group	54.8	31.3	78.2	64.7	21.3	2.2	40.3	25.1
Emergency care costs								
Ambulance costs	23.7	12.5	35.0	28.0	27.5	‐0.8	55.8	32.5
Accident & emergency costs	16.7	9.3	24.0	19.7	22.3	1.1	43.5	26.3
Outpatient care costs								
Non‐MH outpatient care	122.1	89.6	154.7	144.1	142.9	41.8	244.0	168.7
Psychiatrist outpatient care	366.9	327.3	406.5	433.1	313.2	235.5	390.8	369.7
Psychologist outpatient care	34.4	28.1	40.8	40.6	23.1	6.6	39.6	27.3
Hospitalization costs								
Non‐MH inpatient and day‐patient care	513.6	254.3	772.9	606.3	739.5	27.0	1452.0	873.0
MH day‐patient care	222.8	127.5	318.1	263.0	232.8	–70.4	536.1	274.8
MH inpatient care	1225.0	419.7	2030.3	1446.1	415.6	–420.4	1251.6	490.6
Medication costs	859.3	731.8	968.8	1014.4	909.1	559.8	1193.9	1073.2
Social care costs^‡^	59.5	40.3	78.7	68.0	70.8	18.2	123.4	80.9
Out‐of‐pocket costs	111.2	72.3	150.1	131.3	96.8	49.1	144.6	114.3
Lost productivity costs^§^	1554.7	1164.7	1944.6	1843.7	1117.0	461.5	1772.6	1324.6
Informal care costs^§^	7032.9	5318.7	8747.0	8340.3	7159.9	2104.8	12215.0	8490.9
**Total costs**	**12,617**	**10,552**	**14,713**	**14,938**	**11,674**	**5583**	**17,765**	**13,823**

^†^ Price‐adjustments based on the Hospital & Community Health Services (HCHS) until 2014–2015 and the NHS cost Inflation Index (NHSCII) thereafter unless stated otherwise (Curtis & Burns, 2019).

^‡^ Price‐adjustments based on the Personal Social Services (PSS) pay and prices index (Curtis & Burns, 2019).

^§^ Price‐adjustments based on the UK consumer price index (CPI) (ONS, 2020a; ONS 2020b).

### 3.3 Health care costs

Primary care costs included visits to/by general practitioners (GP) and general practice nurses. GP costs accounted for 88% of the total primary care costs. The mean primary care cost per patient was £253 (SE = ±£13, 2018–2019 prices: £299), 2% of the estimated mean annual societal BD cost per patient. The mean annual community mental health care (CMH) cost per patient was £221 (SE = £24, ±2018–2019 prices: £261). These costs were divided among community psychiatric nurse (29%), community psychologists (17%), community psychiatrists (25%), community drop‐in centers (5%) and support group visits (25%). Visits to A&E and use of ambulance services were categorized under emergency care. The mean annual cost of emergency care per patient was £40 (SE = ±£7, 2018–2019 prices: £48), with 59% (£24, 2018–2019 prices: £28) of the costs being associated with the use of ambulance services.

Mental health outpatient care comprises hospital‐based outpatient visits to psychiatrists and psychologists. The mean annual cost of using mental health outpatient services per patient was £401 (SE = ±£23, 2018–2019 prices: £474). Psychiatrist visits accounted for 91% of the total outpatient costs. The mean annual cost of nonmental health outpatient visits per patient was £122 (SE = ±£17, 2018–2019 prices: £144).

Hospitalization costs were analyzed separately for mental health‐related inpatient costs (63%), mental health‐related day‐patient costs (11%), and other, nonmental health inpatient and day‐patient costs (26%). Mean annual mental health‐related inpatient hospitalization costs per patient were estimated at £1225 (SE = ±408, 2018–2019 prices: £1446), mental‐health day‐patient costs at £223 (SE = ±£48, 2018–2019 prices: £263), and nonmental health hospitalization costs at £514 (SE = ±£131, 2018–2019 prices: £606). Overall, hospitalization costs accounted for 16% of the total annual economic burden per patient and formed the largest health care cost subcategory.

### 3.4 Psychiatric medication costs

Mean annual psychiatric medication costs were estimated at £859 (SE = ±£65, 2018–2019 prices: £1014) per patient, the second largest health care cost subcategory contributing to 7% of the total annual economic burden per patient.

### 3.5 Social care costs

Social care costs included services offered by social workers, community support workers, home care workers, housing workers, and voluntary workers. In total, mean annual social care costs per BD patient were estimated to be £60 (SE = ±£10, 2018–2019 prices: £68).

### 3.6 Out‐of‐pocket patient costs

Patient costs were out‐of‐pocket expenditures spent on services offered by private health service providers (e.g., dieticians, chiropodists, private physiotherapists, etc.) and travel costs to mental health facilities. The average annual out‐of‐pocket costs per BD patient were £111 (SE = ±£20, 2018–2019 prices: £131).

### 3.7 Indirect costs (lost productivity and informal care)

Major indirect costs of £8588 (SE = ±£891, 2018–2019 prices: £10,184) occurred due to lost productivity and informal care provided by family members and friends. Informal care costs amounted to £7033 (2018–2019 prices: £8340) and lost productivity due to sickness absence to £1555 (2018–2019 prices: £1844). These costs accounted for 68% of the total annual economic burden of BD per patient in the United Kingdom representing the largest cost component.

### 3.8 Determinants of costs

Results of the GEE analyses are presented in Table 3. Mean annual costs were significantly associated with mean QIDS and ASRM scores over 12 months. A single unit of increase in the QIDS score was associated with 7.7% (*p* = .012) increase in the mean annual societal cost and for a single unit increase in the ASRM score, the increase in mean annual societal costs was 11.6% (*p* = .022). When health care costs were considered, only the association with the severity of mania remained significant at the 5% level; a unit difference in the mean ASRM score was associated with an increase in the mean annual health care costs of 11.5% (*p* = .03). Costs due to lost productivity and informal care were more closely associated with the severity of depression; a single unit of QIDS score increase was associated with an increase in annual indirect costs of 9.2% (*p* = .036).

**TABLE 3:** Determinants of BD costs (MI‐GEE analysis)

Variables	Total costs (1)			Health care costs (2)			Indirect costs (3)		
	Exp (coeff)	Exp (SE)	*p* Value	Exp (coeff)	Exp (SE)	*p* Value	Exp (coeff)	Exp (SE)	*p* Value
BDII	1.041	0.251	.867	1.176	0.363	.599	0.931	0.279	.810
Male	1.016	0.253	.949	0.880	0.244	.645	1.164	0.378	.639
Duration of illness	1.008	0.013	.570	1.008	0.009	.421	1.008	0.018	.638
Mean QIDS score	1.077	0.032	.012^*^	1.066	0.041	.096^+^	1.092	0.046	.036^*^
Mean ASRM score	1.116	0.054	.022^*^	1.115	0.056	.032^*^	1.119	0.069	.066^+^
Time‐points									
6th month	0.743	0.159	.165	0.971	0.222	.899	0.585	0.202	.121
9th month	0.813	0.188	.372	1.087	0.280	.746	0.615	0.218	.170
12th month	0.866	0.191	.513	1.021	0.215	.923	0.752	0.265	.419

The coefficients are the results of MI‐GEE model using Gamma regression with log‐link function.

***p < .001, **p < .01, *p < .05, +*p* < .1.

Time‐points: 3rd month as reference category.

### 3.9 Population‐level economic burden

A total of 913 citations were retrieved following the initial search, out of which, 26 studies were included for abstract review. Eventually, seven studies were deemed as eligible and were included in a random‐effects meta‐analysis to derive a pooled estimated for the 12‐month period prevalence of BD relevant to the United Kingdom for the study period (see Appendix Figure 1).

The characteristics of included studies are provided in Table 4. Prevalence of BD ranged between 0.15% and 3% in individual studies. The pooled annual prevalence estimate of BD was 0.8% (95% CI = 0.43%–1.3%) with high heterogeneity (*I* ^2^ = 96.7%). When applying the prevalence rate of 0.8% to the mid‐2010 and mid‐2018 UK population over 16 years of age (2010: 50.9 million, 2018: 53.8 million), the total number of adults living with BD in the United Kingdom was estimated at 407,567 and 430,491 in 2010 and in 2018, respectively (ONS, 2019).

**TABLE 4:** Studies included for the synthesis of population prevalence estimate of BD in the United Kingdom

Study	Country, year of study	Type of sample, age group	Study population	Diagnostic criteria	Instrument	Diagnosis	Prevalence % (SE)	Type of prevalence
Faravelli et al. (1990)	Italy, Florence, NR	Community, >15 years	Total = 1000 *F* = 537 *M* = 463	DSM‐III, DSM‐III‐R	Structured interview	BD‐I, BD‐II, Cyclothymia	Total = 1.9 (0.3) *F* = 2.8 (0.7) *M* = 0.9 (0.3)	12‐month prevalence
McConnell et al. (2002)	UK, Derry (1993–1994)	Community, 18–64	Total = 923 *F* = 496 *M* = 421	DSM–III–R, ICD‐10	SCAN	BD	Total = 0.2 (0.1)	12‐month prevalence
Ten Have et al. (2002)	Netherlands (1996)	National, general population, 18–64	Total = 7076 *F* = 3488 *M* = 3588	DSM‐III‐R or ICD‐10	CIDI	BD	Total = 1.1 (0.1) *F* = 1.1 (0.2) *M* = 1.1 (0.2)	12‐month prevalence
Scully et al. (2004)	Ireland, Monaghan (1996)	Community, >18 years	Total = 29,542 *F* = 14,563 *M* = 14,979	DSM–III–R	SCID	BD	Total = 0.4 (0.3) *F* = 0.4 (0.05) *M* = 0.3 (0.04)	12‐month prevalence
Jacobi et el. (2004)	Germany (1998–1999)	General population, 18–65	Total = 4181 *F* = 2103 *M* = 2078	DSM‐IV	M‐CIDI	BD	Total = 0.8 (0.1) *F* = 1.1 (0.2) *M* = 0.6 (0.1)	12‐month prevalence
De Graaf et al. (2012)	Netherlands (2007–2009)	National, general population, 18–64	Total = 6646 *F* = 3303 *M* = 3343	DSM‐IV	CIDI‐3	BD	Total = 0.8 (0.2) *F* = 1.0 (0.3) *M* = 0.7 (0.2)	12‐month prevalence
Jacobi et al. (2014)	Germany (2008–2011)	National, general population, 18–79	Total = 5318 *F* = 2778 *M* = 2540	DSM‐IV	M‐CIDI	BD	Total = 1.5 (0.2) *F* = 1.7 (0.3) *M* = 1.3 (0.3)	12‐month prevalence

Using the above prevalence and per patient societal cost estimates, the cost‐of‐illness of BD was estimated at £5.1 billion (£6.4 billion adjusted to 2018–2019). Of these costs, £3.5 billion (£4.4 billion adjusted to 2018–2019) were due to productivity losses and informal care costs, £1.6 billion (£2.0 billion adjusted to 2018–2019) due to health care‐related costs, £24.3 million due to social care costs (£29.3 million adjusted to 2018–2019), and £45.3 million (£56.5 million adjusted to 2018–2019) incurred as patients’ out‐of‐pocket expenses (Table 5).

**TABLE 5:** Economic burden of BD in the United Kingdom (£ million, for years 2010–2011 and 2018–2019)

Cost categories	Cost (2010–2011)	Cost (2018–2019)	% of total
Primary care costs	103.2	128.7	2.0%
Community mental health care costs	90.0	112.3	1.7%
Emergency care costs	16.5	20.5	0.3%
Outpatient care costs	213.3	266.0	4.1%
Hospitalization costs	799.4	996.8	15.5%
Medication costs	350.2	436.7	6.8%
Social care costs	24.3	29.3	0.5%
Out‐of‐pocket costs	45.3	56.5	0.9%
Lost productivity and informal care costs	3500.0	4384.1	68.1%
Total costs	**5142.2**	**6430.8**	**100.0%**

### 3.10 Sensitivity analyses

**FIGURE 2:**
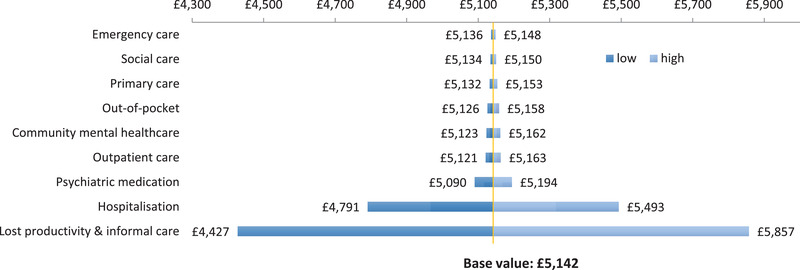
One‐way sensitivity analyses of the estimated economic burden of BD in the United Kingdom according to uncertainty in main cost parameters (in million £s, year 2010–2011)

We conducted a series of one‐way sensitivity analyses and a PSA to test the robustness of the population‐level economic burden result with respect to uncertainty in key parameters for the original costing year 2010–2011. Figure 2 presents variations according to uncertainty in the main cost categories and shows the estimated annual cost‐of‐illness of BD in the United Kingdom ranging between £4.4 billion and £5.9 billion.

Based on specified distributions of all parameters in the PSA, including uncertainty around the population prevalence estimate, the 95% CI of the mean annual societal cost per BD patient was estimated to be between £10,801 and £14,653, while the 95% CI of the annual cost‐of‐illness estimate varied between £2.7 billion and £8.5 billion.

The complete case‐based analysis (Table 2) estimated the total mean annual societal cost per BD patient somewhat reduced at £11,674 lowering the annual cost‐of‐illness estimate to £4.8 billion in the United Kingdom.

## 4 DISCUSSION

The average annual cost of BD per patient was £12,617 in the original year of resource use collection 2010–2011, or £14,938 when adjusted to 2018–2019 prices in our well‐defined cohort of diagnosed BD patients receiving care from specialist mental health services in the United Kingdom. Most of the societal costs (68%) were attributed to lost productivity and informal care costs, 31% to health care costs, 1% to patients’ out‐of‐pocket expenses, and 0.5% to social care costs. The average estimated health care costs of £3859 in 2010–2011 were considerably higher than the Treasury reported average health care expenditure of £1926 per person in the United Kingdom for the same year (HM‐Treasury, 2012). There was an important association between prospectively measured symptom burden over 12 months and health care and societal costs for patients with BD. A unit difference in the mean score for depression (QIDS) was associated with 7% greater total cost; a unit difference in the mania scale (ASRM) was associated with 11% greater total cost. Specifically, costs due to lost productivity and informal care were significantly associated with depression and health care‐related costs with mania. These results are in line with previous studies showing that mania is often associated with increased hospitalizations, while the resource use of individuals with predominantly depressive episodes tends to span across different sectors over a longer period (Begley et al., 2001; Perlis et al., 2006). No association was found between the the type of BD and costs. Our population prevalence synthesis suggested a BD prevalence of around 0.8% in the United Kingdom, resulting in an estimate of the UK adult population aged over 16 years old with BD of 407,567 persons and an overall cost‐of‐illness of £5.1 billion for the year 2010–2011. Adjusting for changes in prices and population, our population‐level economic burden estimate translated to £6.4 billion for 2018–2019.

Overall, our bottom‐up economic burden study confirmed that the majority of the societal costs of BD fall on those who experience the illness and their families, rather than the health care system. Health care expenditure comprises slightly less than a third of overall costs. This is consistent with the existing literature that has highlighted the substantial burden of lost productivity in individuals with BD (Jin & McCrone, 2015; Modini et al., 2016). Individuals with BD consider employment or re‐employment as one of the major elements in their recovery process(Drake et al., 2012). It is, therefore, not only important to investigate the cost‐effectiveness of long‐term management strategies, but also interventions that improve the functioning of individuals who are at work.

Economic burden studies are difficult to compare as they vary in methodology, study setting, and type of costs included. In the United Kingdom, Das Gupta and Guest's (2002) retrospective study used a prevalence estimate of 0.5% adopted from the narrative review of the literature by Bebbington and Ramana (1995), but it was significantly different from the originally reported population‐based estimate of 1%–1.5%. Direct costs in their study accounted for 14% and indirect costs (summarizing costs due to excess unemployment, absenteeism, and suicide) accounted for 86% of total costs in contrast to 31% for direct costs and 68% for costs due to lost productivity and costs for informal care observed in our study. This is likely to be due to the top‐down approach they adopted compared with the bottom‐up approach used in our study. The increased proportion of indirect costs derived in their study was driven by costs assigned to lost employment (85% of indirect costs) followed by costs due to premature mortality (suicides). The estimates for lost employment were obtained from nonpeer‐reviewed reports and assumptions. However, the high proportion of individuals who were unemployed but could work (26%) assumed in Das Gupta and Guest's study (2002) seems to be at odds with much lower rates observed in other relevant studies(Marwaha et al., 2013; McCrone et al., 2008). Moreover, their estimates from 1997 to 1998 included health care resource use of patients who suffered from unipolar psychosis and schizoaffective disorder and this may not be reflective of current practice. Further, we had no observed suicide or other death in our study. Inclusion of mortality‐based production losses incurred in future is generally controversial for prevalence based cost‐of‐illness studies as it does not relate to other time‐defined costs that are usually measured over a year (Jin & McCrone, 2015).

McCrone et al. (2008) modeled societal costs of BD in England by combining health care costs derived from a sample of 103 BD‐I patients participating in a randomized controlled trial (RCT) of cognitive behavioral therapy in England, informal care costs adopted from a psychosis study, and productivity losses adjusted from Das Gupta and Guest's study (2002) along with prevalence data from the United States (Lam et al., 2003; McCrone et al., 2008). Even though, the broad distribution of costs with regard to proportion of direct (31%) and indirect (69%) costs in McCrone et al. (2008) is consistent with our present study, the reported share of hospitalization costs in health care‐related costs (14%) was much lower compared to the result of our present and other similar studies (Ekman et al., 2013; Tafalla et al., 2010; Young et al., 2011). A possible explanation for this is that in assessing service costs, they combined bottom‐up annualized costs based on 3‐month health care resource use for BD‐I patients obtained from a RCT with top‐down estimates of hospitalization (Hospital Episode Statistics data) and residential care for BD‐related conditions. Moreover, for the majority of resource utilization categories, their estimates were derived from a RCT that was undertaken in 1998–1999 with stringent inclusion and exclusion criteria (excluding patients with recent history of active episodes) (Lam et al., 2003). Such samples may not be representative of individuals with BD in the community.

The most recent research assessing costs of BD in the United Kingdom was a top‐down study undertaken by Young et al. (2011), that included only health care costs. They reported that the total hospitalization cost was £207 million corresponding to 60% of all health care costs; this translated to £2370 per BD patient. In comparison, in our present study, hospitalization‐related costs were approximately £799 million for year 2010–2011 accounting for 61% of all health care costs related to BD. Mean hospitalization cost per patient was £1961. The higher average hospitalization costs reported in Young et al. (2011) could be explained by their inclusion of admissions falling under the ICD‐10 code 30.0 which includes manic/hypomanic episodes on its own. Interestingly, in Young et al. only 7% of total health care costs were attributed to medication, while in our present study, we found a share of medication costs in health care costs three times as large (22%).

More importantly, Young et al. (2011) reported a total population‐level health care cost of £342 million (2009–2010), which is much lower than our present study's estimate of £1.6 billion (2010–2011) due to the substantial difference in the prevalence estimate. The prevalence estimate (0.14%) used in their study was based on the number of patients who contacted their GPs in a defined period. This may have underestimated the prevalence in comparison to annual population‐based prevalence data, as likely there are BD patients who do not access psychiatric care within a certain time period.

When comparing our results—converted to US$ PPP using the conversion rates provided by the Organisation for Economic Co‐operation and Development (OECD, 2021)—to the systematic literature review by Kleine‐Budde et al. (2014) for year 2009–2010, we find somewhat lower direct health care costs per patient (US$ PPP 5465) in our study than the range reported by the review not considering outliers (US$ PPP 8000 to US$ PPP 14,000). On the other hand, in line with the studies assessed by Kleine‐Budde et al. (2014), we also find inpatient and medication costs to be the main cost drivers of health care costs. Kleine‐Budde et al. (2014) also reported a high variation of indirect costs between the studies, ranging from US$ PPP 2000 to US$ PPP 11,000. Our mean indirect cost estimate of US$ PPP 12,162 exceeds this range, which is likely due to our prospective individual patient‐level data collection methods on the one hand, and the inclusion of informal care costs (making up for over 80% of indirect costs) on the other hand. A more recent literature review for studies from the United States by Bessonova et al. (2020) reports costs from 56 US studies, with annual all‐cause direct health costs ranging from US$11,239 to US$19,446 for year 2018. While the US estimates are again considerably higher than the direct health care cost of US$ PPP 6663 estimated for the United Kingdom in the present study for year 2018, the variation is well within the expected range with known service and price differences between the United States and the United Kingdom. Bessonova et al. (2020) reported that indirect costs accounted for 72%–79% of the total economic burden of BD in the United States, very similar to our 68% estimate for the United Kingdom. Although some underlying differences between the contributions from unemployment as opposed to sick leave and informal care to the overall level of indirect costs exist between the studies due to methodological reasons, the variations are linkable to known health and social care system differences as well. Overall, the findings of our study are well in line with the large body of international studies on the economic burden of BD when considering the substantial heterogeneity in methods and data types, as well as the numerous differing combinations of cost categories and known system differences.

Further, our present study used True Colours, a method for routine data collection based on patient reported symptoms that is regularly and frequently updated. In an earlier study the system showed high compliance and no impact on mental health service costs except somewhat increased psychiatric medication costs (Simon et al., 2017). Clinical improvement as demonstrated by decreased scores on the QIDS and ASRM even by a single point has statistically significant and financially important impact on annual direct, indirect and overall societal costs. This observed association implies a critical clinical message. More effective care should reduce not only the frequency, severity, and duration of manic and depressive episodes (as measured by QIDS/ASRM scores), but also broader societal economic burden. Repeated patient reported clinical outcomes may also provide a metric for modeling the economic effect of treatment benefits from RCTs.

A final minor point relates to the validity of the ASRM. Self‐rating of mania is often regarded as suspect of bias and potentially confounded by positive mood. In fact, a unit difference in the ASRM was associated with 11% higher illness costs. This implies that the scale does measure a clinically meaningful and impairing effect on health.

### 4.1 Strengths and limitations

This is the first economic burden study that used digitally captured, real‐world, self‐reported population‐based data of BD patients followed‐up over 12 months. This enabled a prospective assessment of clinical status and resource utilization over the course of concomitant enrolment, and the recording and estimation of societal costs for a cohort of patients at four separate occasions without the need for annualizing the cost estimates. Furthermore, the bottom‐up approach allowed the estimation of all relevant costs including health and social care and out‐of‐pocket expenses, and indirect costs (i.e., informal care costs and productivity losses) incurred by individuals with BD. It also enabled the exploration of factors influencing costs using GEE modeling.

Due to the lack of epidemiological studies of BD in the United Kingdom for the year of resource use collection, prevalence estimates were derived from a random‐effects meta‐analysis of population based epidemiological studies of BD in Europe. This yielded a mean prevalence of 0.8% (95% CI = 0.43%–1.3%), which is consistent with a more recently published study that reported the prevalence of BD in the United Kingdom using Bayesian meta‐regression modeling to be 0.8% (95% CI = 0.7%–0.9%) in females and 0.6% (95% CI = 0.5%–0.7%) in males (Ferrari et al., 2016). An estimate of the UK prevalence was made in Adult Psychiatric Morbidity survey of 2014 (Marwaha et al., 2016). Using a relatively crude self‐rated screening instrument, the overall prevalence was 2%, and 0.6% had had the diagnosis confirmed by a health care professional, the cohort most similar to our study. The latter figure is comparable with the European average and illustrates the fact that most patients who screen positive for bipolar disorder are not in active clinical care. By limiting the study to patients with diagnosed and treated BD—as we do in the present study—one is looking only at the tip of the iceberg when considering the burden and cost of bipolar disorder. Taking into consideration the undiagnosed and hence unobserved cases, the complete cost‐of‐illness of BD is likely substantially higher.

We want to caution that our study results need to be interpreted in light of some important limitations. First, the relatively small sample of 91 individuals used in this study was recruited from psychiatric clinics across the Oxfordshire and Buckinghamshire counties in England. While such patients are likely to be more representative than RCT participants, they may not be fully representative of the entire UK BD population. Second, we addressed the presence of missing data with multiple imputation of missing variables assuming a missing at random (MAR) mechanism to limit the loss of too many observations or variables for the analysis. Although there were no statistically significant differences between the baseline characteristics of the complete case patients (*n* = 48) and the overall sample (*n* = 91) and the complete case sensitivity analysis showed no significant impact of data gaps on the overall results, the lower mean costs estimated for the complete cases suggests that patients retained in the follow‐up likely had better functioning outcomes. Third, due to the lack of data, productivity losses for unemployed individuals whose ongoing unemployment was attributed to BD and premature mortality due to BD were not included likely leading to some underestimation of the overall economic burden.

## 5 CONCLUSIONS

We estimate that the total annual cost‐of‐illness for BD amounted to £5.1 billion for the year 2010–2011 (2018–2019: £6.4 billion), resulting in £1.6 billion (2018–2019: £2.0 billion) costs directly related to the health care sector in the United Kingdom. This corresponds to approximately 1.2% of the total government‐financed health care expenditure of £131.0 billion in 2010–2011 (ONS, 2020b). Despite excluding costs attributed to premature mortality, the magnitude of costs reported in our present study mirrors the economic burden of schizophrenia (£6.7 billion) in the United Kingdom (Mangalore & Knapp, 2007). The immense costs of informal care provided by family and friends of BD patients, however, so far have received much less attention than in the case of schizophrenia and dementia. The identification of schizophrenia as the “paradigm condition” or the heartland of psychiatry and the relatively lower burden on health and social care has probably contributed to a relatively low priority for research into BD and resulting low research spend (Harrison et al., 2016).

The magnitude of health care and social care‐related costs and the costs of informal care and due to lost productivity were significantly associated with self‐reported levels of mood symptoms. From a public mental health perspective, these results generate the hypothesis that significant cost savings could result from improved monitoring and control of self‐reported symptoms between episodes using a low‐cost and relatively simple method of mood monitoring.

This study provides robust evidence that BD is a disease of substantial economic burden in the United Kingdom. Policy makers need to pay attention to this distribution of expenditure in BD as health care and medications represent a lesser proportion of the total economic burden, implying that the focus of any future interventions should be related to improving the functionality and productivity of individuals with BD.

## Figures and Tables

**FIGURE A1:**
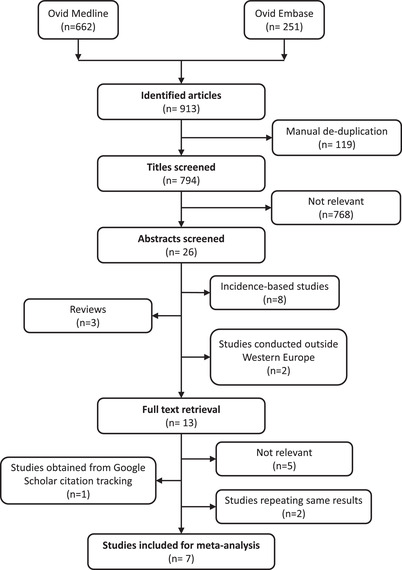
Literature search for population prevalence estimates of BD in Europe (PRISMA chart)

## Data Availability

Data sharing was not mandated for the OXTEXT programme (2009‐2015).

## 1

**TABLE A1:** Published UK economic burden of BD studies

Study	Year of original study and study perspective	Type and perspective of study	Estimated prevalence, *n* (%)	Data source	Average per person health care costs	Average per person societal costs	Total health care costs (NHS perspective)	Cost‐of‐illness	Cost of hospitalizations proportional to health care costs	Proportion of COI attributed to indirect costs
Das Gupta and Guest, 2002	1997–1998 societal	Top‐down; prevalence‐based	297,000 (0.5%)	DIN‐link database, HES	£670	£6919	£199 million	£2 billion	50%	86%
McCrone et al., 2008	1998–1999 societal	Mixture of top‐down & bottom‐up; prevalence‐based	1.14 million (2.2%)	Report based on published literature	£1404	£4561	£1.6 billion	£5.2 billion	15%	69%
Young et al., 2011	2007–2008 health care	Top‐down; prevalence‐based	87,338 (0.14%)	IMS Disease Analyser, HES, MHMDS	£3916	NA	£342 million	NA	60%	NA

HES, hospital episode statistics; IMS Disease Analyser, database of anonymized disease and therapy pathways provided by IMS Health; MHMDS, mental health minimum.

Dataset Statistics.

**TABLE A2:** Unit costs for resource use (£, 2010–2011)

Resource use	Unit costs		Source of estimate
**Nonmental health in‐patient services**			
Nonmental health inpatient admissions	£58–£1610	Per day	Curtis & Netten (2010), NHS reference costs (2010–2011)
**Nonmental health out‐patient & emergency services**			
Outpatient	£54–£171	Per visit	NHS reference costs (2010–2011)
Laboratory investigations	£3‐£177	Per test	NHS reference costs (2010–2011)
Diagnostic imaging	£29–£173	Per test	NHS reference costs (2010–2011)
A & E	£107	Per visit	NHS reference costs (2010–2011)
Ambulance	£220	Per call‐out	NHS reference costs (2010–2011)
**Mental health out‐patient**			
Psychiatrist	£3.00	Per minute	NHS reference costs (2010–2011)
Clinical psychologist	£0.60	Per minute	Curtis & Netten (2010)
**Mental health in‐patient**			
Psychiatry	£327	Per night	NHS reference costs (2010–2011)
Psychiatric intensive care unit (PICU)	£655	Per night	NHS reference costs (2010–2011)
Mental health day‐care	£21–127	Per session‐per day	Curtis & Netten (2010) & NHS reference costs (2010–2011)
**Community‐based services**			
Community psychiatrist	£1.85	Per minute	NHS reference costs (2010–2011)
Community psychologist	£0.60	Per minute	Curtis & Netten (2010)
Community psychiatry nurse	£0.50	Per minute	Curtis & Netten (2010)
Community support worker	£0.25	Per minute	Curtis & Netten (2010)
Drug and alcohol services	£50.00	Per contact	NHS reference costs (2010–2011)
GP	£32.00	Per consultation	Curtis & Netten (2010)
GP practice nurse	£12.00	Per consultation	Curtis & Netten (2010)
Home care worker	£0.40	Per minute	Curtis & Netten (2010)
Housing worker	£0.26	Per minute	assuming national average salary, ONS (2010–2011)
Social worker	£0.88	Per minute	Curtis & Netten (2010)
Voluntary worker	£0.26	Per minute	assuming national average salary, ONS (2010–2011)
Other services	£8.8–£192		Curtis & Netten (2010) & NHS reference costs (2010–2011)
**Other**			
Sick leave	£83.00	Per day	NICE & Sainsbury Mental Health Centre 2010–2011
Informal care	£15.6	Per hour	assuming national average salary, ONS (2010–2011)
